# Progress in Surgery

**Published:** 1899-10-28

**Authors:** 


### Progress in Surgery.

### SURGERY OF THE NERVES.

**Operations on the Sympathetic Nervous System.**—Since Jonnesco and others some years ago showed the possibility of removing the various sympathetic ganglia in the neck, and demonstrated the improvement which followed therefrom in certain cases of epilepsy and Graves' disease, others have followed in their track, and have extended the principle into other regions and against different diseases. Thus Ruggi[1] has removed the superior cervical ganglion in ten cases of glaucoma with a view to paralysing the vaso-dilator nerves which take origin from the sympathetic cord. In some cases the operation was performed on both sides, in others only on one. In most of the cases pain was markedly relieved, temporarily at least, but sufficient time has not yet elapsed to determine the other effects of the procedure.

The same surgeon has also removed the utero-ovarian plexus for severe pelvic pain which persisted after removal of the ovaries. In three such cases the disappearance of the pain was speedy, and, so far, permanent. In a fourth case extreme uterine tenderness was immediately cured by removal of a portion of the sympathetic plexus in the region of the pylorus.

Deschamps[2] has recently operated upon two young patients suffering from epilepsy by removing the superior cervical ganglion, an operation which he considers both difficult and dangerous. One of the patients was a microcephalic child; in the other case the fits were of traumatic origin. The record of these cases, however, is practically valueless as regards the results, as only thirteen and eight days respectively had elapsed between the operation and the publication of the cases. All that they prove is that the removal is possible, and not necessarily immediately fatal.

**Surgery of the Peripheral Nerves.**—A successful case of nerve transplantation is reported by Peterson.[3] He used the sciatic nerves of a dog to fill up the gap between the divided ends of the median and ulnar nerves of one of his patients. Three months after the operation the hand, which had been paralysed and anæsthetic over the whole distribution of these trunks, had completely regained its muscular power, and nearly all its sensation. The author has collected the records of 19 other similar cases, and from their study concludes that this procedure is a useful and justifiable one, being followed in some cases by complete, or in others by at least partial restoration of function. The reparative process is slow, so that patience must be exercised in waiting for results. Sensation as a rule returns before motion, probably because of a collateral nerve supply.

A case of resection of the genito-crural nerve for severe ilio-scrotal pain is reported by Donath and Hültl.[4] Following an attack of gonorrhœa with left-sided epidymitis the patient had severe pain, which for four years had rendered life a burden to him. He was desirous of having the testes removed, but the depressing effects of this operation and the uncertainty of cure following led the authors to resect the genito-crural nerve. This was done through a long curved incision from the eleventh rib to the middle of Poupart's ligament. The peritoneum was stripped back without being opened until the common iliac artery was exposed, and, the genital and crural branches of the nerve having been isolated, about 2½ inches of each was resected. Primary union took place, and the pain disappeared for about two months, when it returned in a much less aggravated degree. There was no lesion discoverable in the portions of nerve removed, which, together with the recurrence of pain, tends to support the view that the lesion is in the spinal cord or in the sympathetic.

**Injuries to Peripheral Nerves.**—Horsley[5] lays stress on the importance of bearing in mind all the constituent elements—motor, sensory, vaso-motor, and trophic fibres—of a mixed nerve in relation to injuries, as we may have interference with any or all these elements, giving rise to clinical manifestations. In the regeneration of divided nerve fibres the axis cylinders of the proximal end grow down into the peripheral end and re-establish function. This may take a very long time, up to two or three years to be complete, and intervals of nearly two inches may be bridged over. Mr. Horsley is of opinion that many surgeons operate too early in these cases before nature has had a fair chance to do her best. Peripheral nerves are liable to be cut, contused, compressed by callus, or stretched by violence. The last-named injury is most apt to occur in the sciatic and cervical nerve trunks. The fifth and sixth cervical nerves when stretched give rise to paralysis of Erb's group of muscles—the deltoid biceps and supinator longus; and the extensors of the wrist and of the fingers. Although motor paralysis is severe, the sensory disturbance is slight. Pain is a prominent symptom in all forms of injury, but its value in localising the seat of the lesion, or its extent, is not great. Vaso-motor paralysis, with patches of hyperæmia and œdematous swelling, is an important symptom.

After operation sensation often returns almost at once, but soon disappears again, and returns in the course of a few weeks. The prognosis in all cases is good, but great patience must be exercised, as it may be as long as five or six years before recovery is complete. Traumatic neurasthenia often complicates these cases. The paralysis due to motor nerve interference and that due to neurasthenia may be distinguished clinically by grasping the affected muscle between the hands, and noting that when the patient makes a voluntary effort, in the former case the muscular belly remains soft and flaccid, while in the latter it rises in contraction, although its power is slight. Sensory stimuli also are incorrectly located by a neurasthenic patient.

**Subcutaneous Injury to the Peroneal Nerve.**—Two cases in which the peroneal nerve was severely contused, leading to its complete paralysis, are reported by Hogarth.[6] As no improvement took place at the end of about three months, operation was performed and the nerve exposed and freed from adhesions, with satisfactory results in both cases. In commenting on these cases the author remarks that severe injuries to nerves without division of skin are not common. The external popliteal nerve, on account of its superficial position and its close relation to the fibula, is specially liable to contusion. In both his cases the paralysis was due to pressure of cicatrix and not to division of fibres, a point which it is exceedingly difficult to determine without operation. Sensation always returns more rapidly and more completely than muscular power. As long as the paralysed muscles react to the galvanic current the operation is worth trying. If they do not so react the only benefit to be hoped for from operation is a return of sensation. Asepsis is the all important factor in operating. So soon as voluntary power begins to return the patient should be encouraged to use the muscles.

**Removal of the Gasserian Ganglion.**—Erdmann[7] reports a new case of the Hartley-Krause operation for trifacial neuralgia, in a man 53 years of age, who had all the usual symptoms to an acute degree. The operation was at once followed by cessation of pain. Seven weeks afterwards corneal ulceration set in in spite of ordinary precautions having been taken.

1 Il Policlinico, May 15, 1899; Brit. Med. Jour. (Supp.), Aug. 26, 1899.

2 Gaz. Méd. Belge, No. 22, 1899; Brit. Med. Jour. (Supp.) July 22, 1899.

3 Amer. Jour. Med. Sci., April, 1899. 4 Wien. Klin. Wochen, Mar., 1899.

5 Practitioner, Aug., 1899. 6 Lancet, July 29, 1899. 7 New York Med. Jour., May, 1899.

